# Multicenter study of skin rashes and hepatotoxicity in antiretroviral-naïve HIV-positive patients receiving non-nucleoside reverse-transcriptase inhibitor plus nucleoside reverse-transcriptase inhibitors in Taiwan

**DOI:** 10.1371/journal.pone.0171596

**Published:** 2017-02-21

**Authors:** Pei-Ying Wu, Chien-Yu Cheng, Chun-Eng Liu, Yi-Chien Lee, Chia-Jui Yang, Mao-Song Tsai, Shu-Hsing Cheng, Shih-Ping Lin, De-Yu Lin, Ning-Chi Wang, Yi-Chieh Lee, Hsin-Yun Sun, Hung-Jen Tang, Chien-Ching Hung

**Affiliations:** 1 Center of Infection Control, National Taiwan University Hospital, Taipei, Taiwan; 2 Department of Internal Medicine, Taoyuan General Hospital, Ministry of Health and Welfare, Tao-Yuan, Taiwan; 3 School of Public Health, National Yang-Ming University, Taipei, Taiwan; 4 Department of Internal Medicine, Changhua Christian Hospital, Changhua, Taiwan; 5 Department of Internal Medicine, Ditmanson Medical Foundation Chia-Yi Christian Hospital, Chia-Yi, Taiwan; 6 School of Medicine, National Yang-Ming University, Taipei, Taiwan; 7 Department of Internal Medicine, Far Eastern Memorial Hospital, New Taipei City, Taiwan; 8 School of Public Health, College of Public Health and Nutrition, Taipei Medical University, Taipei, Taiwan; 9 Department of Internal Medicine, Taichung Veterans General Hospital, Taichung, Taiwan; 10 Department of Internal Medicine, Tri-Service General Hospital and National Defense Medical College, Taipei, Taiwan; 11 Department of Internal Medicine, Lotung Poh-Ai Hospital, Medical Lo-Hsu Foundation, I-Lan, Taiwan; 12 Department of Internal Medicine, National Taiwan University Hospital and National Taiwan University College of Medicine, Taipei, Taiwan; 13 Department of Internal Medicine, Chi Mei Medical Center, Tainan, Taiwan; 14 Department of Health and Nutrition, Chia Nan University of Pharmacy and Sciences, Tainan, Taiwan; 15 Department of Parasitology, National Taiwan University College of Medicine, Taipei, Taiwan; Universidad Autonoma de Madrid Centro de Biologia Molecular Severo Ochoa, SPAIN

## Abstract

**Objectives:**

Two nucleos(*t*)ide reverse-transcriptase inhibitors (NRTIs) plus 1 non-NRTI (nNRTI) remain the preferred or alternative combination antiretroviral therapy (cART) for antiretroviral-naive HIV-positive patients in Taiwan. The three most commonly used nNRTIs are nevirapine (NVP), efavirenz (EFV) and rilpivirine (RPV). This study aimed to determine the incidences of hepatotoxicity and skin rashes within 4 weeks of initiation of cART containing 1 nNRTI plus 2 NRTIs.

**Methods:**

Between June, 2012 and November, 2015, all antiretroviral-naive HIV-positive adult patients initiating nNRTI-containing cART at 8 designated hospitals for HIV care were included in this retrospective observational study. According to the national HIV treatment guidelines, patients were assessed at baseline, 2 and 4 weeks of cART initiation, and subsequently every 8 to 12 weeks. Plasma HIV RNA load, CD4 cell count and aminotransferases were determined. The toxicity grading scale of the Division of AIDS (DAIDS) 2014 was used for reporting clinical and laboratory adverse events.

**Results:**

During the 3.5-year study period, 2,341 patients initiated nNRTI-containing cART: NVP in 629 patients, EFV 1,363 patients, and RPV 349 patients. Rash of any grade occurred in 14.1% (n = 331) of the patients. In multiple logistic regression analysis, baseline CD4 cell counts (per 100-cell/μl increase, adjusted odds ratio [AOR], 1.125; 95% confidence interval [95% CI], 1.031–1.228) and use of NVP (AOR, 2.443; 95% CI, 1.816–3.286) (compared with efavirenz) were independently associated with the development of skin rashes. Among the 1,455 patients (62.2%) with aminotransferase data both at baseline and week 4, 72 (4.9%) developed grade 2 or greater hepatotoxicity. In multiple logistic regression analysis, presence of antibody for hepatitis C virus (HCV) (AOR, 2.865; 95% CI, 1.439–5.704) or hepatitis B surface antigen (AOR, 2.397; 95% CI, 1.150–4.997), and development of skin rashes (AOR, 2.811; 95% CI, 1.051–7.521) were independently associated with the development of hepatotoxicity.

**Conclusions:**

The baseline CD4 cell counts and use of NVP were associated with increased risk of skin rashes, while hepatotoxicity was independently associated with HCV or hepatitis B virus coinfection, and development of skin rashes in antiretroviral-naïve HIV-positive Taiwanese patients within 4 weeks of initiation of nNRTI-containing regimens.

## Introduction

In recent practice guidelines of first-line antiretroviral treatment of HIV infection, the preferred or alternative combination antiretroviral therapy (cART) regimens include a combination of two nucleo(*t*)side reverse-transcriptase inhibitors (NRTIs) (tenofovir disoproxil fumarate [TDF] and emtricitabine or lamivudine) plus an active drug from one of the following classes: integrase strand transfer inhibitor (INSTI), ritonavir-boosted protease inhibitor (PI) [1–3], non-nucleoside reverse transcriptase inhibitor (nNRTI) (efavirenz [EFV] [2] or rilpivirine [RPV])[3]. The World Health Organization (WHO) Guidelines 2015 recommend either nevirapine (NVP) or EFV as a part of first-line antiretroviral therapy [4]. Other than efficacy, the choice of first-line therapy is determined based on various considerations, which include safety, drug tolerability, transmission of drug-resistant HIV-1 in the untreated population, coinfections, such as tuberculosis [5] and viral hepatitis, pregnancy, comorbidities, concurrent medications, or availability of antiretroviral agents. The cost of antiretroviral therapy is also an important factor to consider, especially in countries with limited resources [6].

The antiretroviral regimens containing the first-generation nNRTIs, EFV and NVP, have been shown to be efficacious and safety in different populations [7, 8]. In patients co-infected with HIV infection and tuberculosis, EFV remains the preferred nNRTI to be combined with rifampicin-containing anti-tuberculous therapy [5, 9]; however, neuropsychiatric symptoms are common adverse effects of EFV [10, 11]. In contrast, NVP is the preferred nNRTI in the first-line antiretroviral regiments in pregnancy because of substantial clinical experience in pregnant women and its proven efficacy in reducing mother-to-child transmission [12, 13]; however, higher incidences of rash, Stevens-Johnson syndrome, and hepatotoxicity have been associated with NVP than EFV [7, 14–17].

The frequency of elevation of liver enzymes in patients on EFV-containing regimens ranges from 1 to 8% [7, 8, 18–20], whereas in patients treated with NVP-containing regimens, it ranges from 4 to 18% [8, 16, 18–22]. NVP-related hepatotoxicity occurs almost exclusively during the first 6 weeks of treatment, which is more likely to develop in women with CD4 cell counts >250 cells/μl and in men with CD4 cell counts >400 cells/μl [23, 24], In previous studies, the associated factors with EFV-related hepatotoxicity were hepatitis C virus (HCV) coinfection and excessive alcohol use [25, 26]; moreover, skin rash has been reported to be associated with symptomatic hepatitis [23].

The second-generation nNRTIs, RPV, has demonstrated antiviral efficacy similar to that of EFV in antiretroviral-naïve adults with baseline plasma HIV RNA load (PVL) ≦100,000 copies/ml over 96 weeks in phase 3 clinical trials (ECHO and THRIVE) [27–29]. Compared with EFV, RPV was associated with a significantly lower incidence of skin rash (4% vs. 9%) and treatment-emergent elevation of aminotransferase levels (6% vs. 10–11%) [28].

In Taiwan, the three most commonly used nNRTIs among antiretroviral-naive patients are NVP, EFV, and RPV. A higher prevalence of chronic viral hepatitis B or C among HIV-positive patients in Taiwan [30, 31] and pharmacokinetics of antiretroviral therapy [32] has raised our concerns about the potential risks of hepatotoxicity and skin rashes related to the use of nNRTIs as the first-line antiretroviral therapy [33]. This multicenter, retrospective observational study aimed to investigate the incidences of skin rashes and hepatotoxicity within the first 4 weeks of initiation of nNRTI-containing antiretroviral therapy in HIV-1-infected adult patients in Taiwan.

## Methods

### Study population and setting

This retrospective observational study was conducted at 8 designated hospitals for HIV care around Taiwan (National Taiwan University Hospital, Tri-Service General Hospital, Far Eastern Memorial Hospital, Taoyuan General Hospital, Taichung Veterans General Hospital, Changhua Christian Hospital, Chia-Yi Christian Hospital and Chi Mei Hospital). We included all HIV-positive patients aged 20 years or greater who were antiretroviral-naive and initiated nNRTI-containing cART between 1 June, 2012 and 31 November 2015. All patients were followed until 31 January, 2016, death or loss to follow-up, whichever occurred first. The study was approved by the research ethics committee of each participating hospital and informed consent was waived.

HIV care, including cART and monitoring of CD4 cell count and PVL, has been provided free-of-charge since cART became available in Taiwan in April 1997. Due to financial constraints on the provision of free-of-charge access to cART, the Centers of Disease Controls (CDC) in Taiwan implemented regulations on the prescription of cART to antiretroviral-naive HIV-positive patients who received their first-line cART on 1 June 2012. Four categories of cART were defined: the first category consisted of NVP, RPV or EFV plus zidovudine/lamivudine (coformulated); the second category, NVP or EFV plus abacavir/lamivudine (coformulated); or TDF/emtricitabine (coformulated) or TDF and lamivudine; the third category, zidovudine/lamivudine plus protease inhibitors (PIs) or raltegravir; and the fourth category, TDF/emtricitabine, TDF and lamivudine, or abacavir/lamivudine plus PIs or raltegravir. Patients could start antiretroviral regimens in the first three categories, but initiation of regimens in the fourth category required approval before prescription. Raltegravir was not available in clinical use for antiretroviral-experienced patients until 2009; and in 2012, it was available for antiretroviral-naïve patients to be combined with 2 NRTIs. RPV was not available until January 2014. In patients with chronic HBV infection, TDF-containing regimens were recommended and RPV was recommended only for patients with baseline PVL <100,000 copies/ml.

### Laboratory investigations

Before initiation of cART, baseline assessment included hemogram, CD4 count, PVL, serologic markers of syphilis and hepatitis A, B, and C viruses, urinalysis, and serum biochemistry, including total bilirubin, alanine aminotransferase (ALT), aspartate aminotransferase (AST) and lipid profiles. Transmitted drug resistance mutations of HIV-1 to NRTIs, nNRTIs, PIs, and INSTIs were not routinely determined before cART was initiated; genotypic resistance testing was only performed retrospectively for the purposes of surveillance [34, 35].

After initiation of cART, patients were mandatorily enrolled in case management program implemented by Taiwan CDC and patients were usually seen 2 weeks after initiation of cART to assess the adverse effects and tolerability of the regimens prescribed. Aminotransferases and hemogram were determined. Patients returned for reassessment of virological and immunological responses and adverse effects at week 4 of cART, and subsequently every 8 to 12 weeks. At these visits, physical examination was performed and PVL, CD4 cell count, serum chemistries, including total bilirubin, AST, ALT, and lipid profiles, were determined to assess the clinical and laboratory adverse events. A standardized case record form was used to collect information on demographic and clinical characteristics and immunological and virological responses.

### Definitions

We assessed the incidence of skin rashes within 4 weeks of cART initiation. Hepatotoxicity grading was based on ALT and AST levels, which was defined in accordance with the Division of AIDS Table for Grading the Severity of Adult and Pediatric Adverse Events (DAIDS AE Grading Table) [36], in the following manner: grade 1, 1.25–2.4 times the upper limit of normal (ULN) (upper normal values, 31–41 U/L for AST and 41–44 U/L for ALT, depending on the ULN values of each participating hospital); grade 2, 2.5–4.9 × ULN; grade 3, 5.0–9.9 × ULN; and grade 4, ≧10 × ULN, for those patients with normal aminotransferase levels at baseline. For patients with abnormal aminotransferase levels at baseline, hepatotoxicity was defined as a 2-fold or greater increase from baseline levels [33]. The skin rashes was graded according to the Division of AIDS Table for Grading the Severity of Adult and Pediatric Adverse Events (DAIDS AE Grading Table) [36].

HCV infection was defined as presence of antibodies for HCV, while hepatitis B virus (HBV) infection was defined as presence of HBV surface antigen (HBsAg).

### Statistical analysis

Categorical variables were analyzed by using X^2^ test and continuous variables were compared using Student’s *t* test. A *P*-value of <0.05 was considered statistically significant. All *P* values were two-tailed. Variables with *P*<0.10 or those of biological significance in the univariate analyses were entered into a multivariate logistic analyses. Association between hepatotoxicity and skin rash and clinical characteristics were assessed in logistic regression analysis. Variables included in these analyses were age, gender, HIV risk group, baseline CD4 cell count, baseline PVL, and baseline AST and ALT. Statistical analyses were performed using SAS software (Version 9.3).

## Results

### Clinical characteristics of the patients

A total of 2,341 antiretroviral-naïve patients started cART between June 2012 and November 2015; 629 patients received NVP plus 2 NRTIs, 1,363 patients EFV plus 2 NRTIs, and 349 patients RPV plus 2 NRTIs. Their baseline demographic and clinical characteristics are shown in Table 1. Overall, the great majority (95.8%) of the patients were male, with a mean age of 33 years, and men who have sex with men and injection drug users accounted for 77.7% and 16.7% of the patients, respectively. While the mean PVL was 4.7 log_10_ copies/mL, most of the patients initiated cART late with a mean CD4 count 279 cells/μl and only 29.8% initiated cART with a baseline CD4 count of 350 cells/μl or greater (data not shown). HBsAg was determined in 2,291 patients (97.9%) with 275 (12.0%) testing positive, while anti-HCV antibody was determined in 2,286 patients (97.7%) with 437 (19.1%) testing positive.

**Table 1:** Clinical characteristics of the patients initiating non-nucleoside reverse-transcriptase inhibitor-containing regimens.

Variable	ALL	NVP group	EFV group	RPV group	P*
Case number, n (%)	2341	629	1363	349	-
Age, mean (SD), years	33 (9.3)	33 (10)	33 (9.3)	33 (8.6)	0.7905
Male sex, n (%)	2242 (95.8)	597 (94.9)	1314 (96.4)	331 (94.8)	0.1979
Risk behavior for HIV transmission, n (%)					0.0025
MSM	1818 (77.7)	493 (78.4)	1080 (79.2)	245 (70.2)	
IDU	390 (16.7)	96 (15.2)	209 (15.3)	85 (24.3)	
Others	133 (5.7)	40 (6.4)	74 (5.4)	19 (5.4)	
HBsAg positivity, n (%)	275/2291 (12.0)	56/617 (9.1)	202/1333 (15.2)	17/341 (5.0)	< .0001
Anti-HCV positivity, n (%)	437/2286 (19.1)	99/618 (16.0)	243/1331(18.3)	95/337 (28.2)	< .0001
CD4 count at baseline, mean (SD), cells/μl	279 (183)	214 (127)	281 (189)	388 (194)	< .0001
Plasma HIV RNA load at baseline, mean (SD), log_10_ copies/ml	4.7 (0.8)	4.8 (0.8)	4.8 (0.8)	4.3 (0.6)	< .0001
Baseline AST, mean (SD), IU/L	42 (84)	38 (98)	45 (86)	37 (38)	0.0398
Baseline ALT, mean (SD), IU/L	43 (97)	35 (43)	46 (120)	42 (59)	0.0010
NRTIs, n (%)					
ZDV/3TC	1219 (52.1)	355 (56.4)	537 (39.4)	327 (93.7)	< .0001
ABC/3TC	130 (5.6)	45 (7.2)	82 (6.0)	3 (0.9)	0.0001
TDF/3TC or TDF/FTC	986 (42.1)	224 (35.6)	743 (54.5)	19 (5.4)	< .0001
CD4 count at 1 month, mean (SD), cells/μl,	398 (222)	333 (177)	401 (224)	491 (241)	< .0001
Plasma HIV RNA load at 1 month, mean (SD), log_10_ copies/ml,	2.5 (0.8)	2.7 (0.9)	2.5 (0.8)	2.2 (0.7)	< .0001

*P value was calculated for the differences among the three groups. Continuous variables were analyzed with nonparametric statistics, Kruskal-Wallis H test, while categorical variables with chi-square test.

**Abbreviations:** 3TC, lamivudine; ABC, abacavir; ALT, alanine aminotransferase; AST, aspartate aminotransferase; EFV, efavirenz; FTC, emtricitabine; HBsAg, hepatitis B virus surface antigen; HCV, hepatitis C virus; IDU, injection drug users; MSM, men who have sex with men; NVP, nevirapine; RPV, rilpivirine; SD, standard deviation; TDF, tenofovir disoproxil fumarate; ZDV, zidovudine.

Not unexpectedly, clinical characteristics differed significantly among the patients initiating cART with 3 different regimens consisting of nNRTIs plus 2 NRTIs because of the regulations on prescription of the first-line antiretroviral therapy in antiretroviral-naïve patients, a higher prevalence of chronic HBV infection that required cART containing TDF, and injection drug users who often had low PVL and higher CD4 counts at baseline than other risk groups owing to infections with defective HIV-1 subtype CRF 07_BC in Taiwan [37]. Therefore, a higher proportion of patients who initiated RPV plus 2NRTIs were injection drug users than those who initiated EFV or NVP plus 2 NRTIs (24.3% vs 15.3% and 15.2%, respectively) (Table 1). Compared with patients initiating EFV or NVP plus 2 NRTIs, patients initiating RPV plus 2 NRTIs had a lower mean PVL (4.3 vs 4.8 log_10_ copies/ml) and higher CD4 count (388 vs 281 and 214 cells/μl, respectively).

Within the first 4 weeks of cART, the percentage of patients who discontinued nNRTIs due to any adverse events was 55.4%. Of the patients discontinuing nNRTIs, 67.1% were due to skin rash, 23.2% due to neuropsychiatric symptoms, 5.6% due to gastrointestinal upset, 5.3% due to hepatitis, and 1.1% due to depression.

### Skin rashes: Incidence and associated factors

Within 4 weeks of cART, rash of any grade occurred in 331 (14.1%) of the patients: 149 (23.7%) in NVP group, 180 (13.2%) in EFV group, and 2 (0.6%) in RPV group. The mean interval between initiation of cART and development of skin rashes was longer for the NVP group than EFV group (22 vs 16 days, *P* = 0002). Of the 149 patients who received NVP with skin rash, the proportions of HBV coinfection did not differ between those who discontinued and those who continued NVP (10.6% vs. 14.3%, p = 0.756), and neither did the proportions of HCV coinfection (19.2% vs. 14.3%, P = 0.749). Likewise, of the 180 patients who received EFV with rash, the proportions of HBV coinfection did not differ between those who discontinued and those who continued EFV (10.6% vs. 25.0%, p = 0.131), and neither did the proportions of HCV coinfection (14.2% vs. 25.0%, P = 0.311) (data not shown).

Table 2 shows the results of univariate analyses of factors associated with skin rash for all patients after initiation of nNRTI-containing regimens within the first 4 weeks. In univariate analysis, patients who initiated NVP plus 2 NRTIs had a higher risk of developing skin rashes (*P*<0.0001), while those who initiated RPV plus 2 NRTIs had a lower risk. In multiple logistic regression analysis among patients who received NVP or EFV, we found a higher baseline CD4 cell counts (per 100-cell/μl increase, adjusted odds ratio [AOR], 1.125; 95% confidence interval [95% CI], 1.031–1.228) and use of NVP plus 2 NRTIs (AOR, 2.443; 95% CI, 1.816–3.286) were independently associated with the development of skin rashes (Table 3).

**Table 2:** Univariate analyses for factors associated with skin rash after initiation of nNRTI-containing regimens within the first 4 weeks.

Variable	Skin rash	No skin rash	All	P
	n = 331	n = 2010	n = 2341	
Age, mean (SD), years	33.21 (9.55)	33.36 (9.27)	33.34 (9.31)	0.7805
Gender, male, n (%)	314 (94.86)	1928 (95.92)	2242 (95.77)	0.3762
Baseline CD4, mean (SD), cells/μl	274.6 (177.3)	279.5 (184.5)	278.8 (183.5)	0.6503
Baseline CD4 ≥200 cells/μl, n (%)	218 (66.67)	1304 (66.06)	1522 (66.15)	0.8296
Baseline CD4 ≥250	176 (53.82)	1080 (54.71)	1256 (54.58)	0.7650
Baseline CD4 ≥350	97 (29.66)	591 (29.94)	688 (29.90)	0.9197
Baseline PVL, mean (SD), log_10_ copies/ml	4.80 (0.83)	4.73 (0.78)	4.74 (0.78)	0.1630
HBsAg-positive, n (%), [n = 2291]	36 (11.11)	239 (12.15)	275 (12.00)	0.5938
Anti-HCV-positive, n (%), [n = 2286]	54 (16.67)	383 (19.52)	437 (19.12)	0.2261
Baseline AST, mean (SD), IU/L,	39.7 (59.6)	42.4 (87.9)	41.9 (84.3)	0.5175
Baseline ALT, mean (SD), IU/L,	41.4 (67.3)	43.1 (101.3)	42.8 (97.1)	0.7229
NVP, n (%)	149 (45.02)	480 (23.88)	629 (26.87)	< .0001
EFV, n (%)	180 (54.38)	1183 (58.86)	1363 (58.22)	0.1261
RPV, n (%)	2 (0.60)	347 (17.26)	349 (14.91)	< .0001

**Abbreviations:** 95% CI, 95% confidence interval; ALT, alanine aminotransferase; AST, aspartate aminotransferase; HBsAg, hepatitis B surface antigen; HCV, hepatitis C virus; nNRTI, non-nucleoside reverse-transcriptase inhibitor; NVP, nevirapine; RPV, rilpivirine; SD, standard deviation.

**Table 3:** Multivariate analyses for factors associated with skin rash after initiation of nNRTI-containing regimens within the first 4 weeks (patients receiving rilpivirine were excluded).

Variable	OR*	95%CI*
Age	1.004	0.988–1.020
Male gender	0.691	0.359–1.329
Baseline CD4 cells/μl, per 100-cell/μl increase	1.125	1.031–1.228
Baseline PVL log_10_ copies/m	1.083	0.893–1.315
HBsAg-positive	0.949	0.626–1.438
Anti-HCV-positive	0.842	0.568–1.247
Baseline AST, per 1-IU/L increase	0.999	0.997–1.002
Baseline ALT, per 1-IU/L increase	1.000	0.998–1.002
NVP (vs EFV)	2.443	1.816–3.286

* These analyses were conducted in 1992 patients.

**Abbreviations:** 95% CI, 95% confidence interval; ALT, alanine aminotransferase; AST, aspartate aminotransferase; EFV, efavirenz.

HBsAg, hepatitis B surface antigen; HCV, hepatitis C virus; nNRTI, non-nucleoside reverse-transcriptase inhibitor; NVP, nevirapine; PVL, plasma HIV RNA load.

For each of nNRTI-containing regimen, the results of multivariate analysis of associated factors with skin rashes are shown in S1 Table. In NVP group, we found that baseline CD4 cell count (*P* = 0.05) and age (*P* = 0.04) were associated with developing skin rashes in univariate analysis (data not shown), while in multiple logistic regression analysis, we were not able to identify any factor statistically significantly associated with developing skin rashes. In EFV group, developing skin rashes was associated with older age (*P* = 0.02) and baseline CD4 cell count≧350 cells/μl (*P* = 0.004) in univariate analysis (data not shown). In multiple logistic regression analysis, only baseline CD4 cell count ≧350 cells/μl (AOR, 2.326; 95% CI, 1.211–4.466) was independently associated with the development of skin rashes.

### Hepatotoxicity: Incidence and associated factors

**Fig 1:**
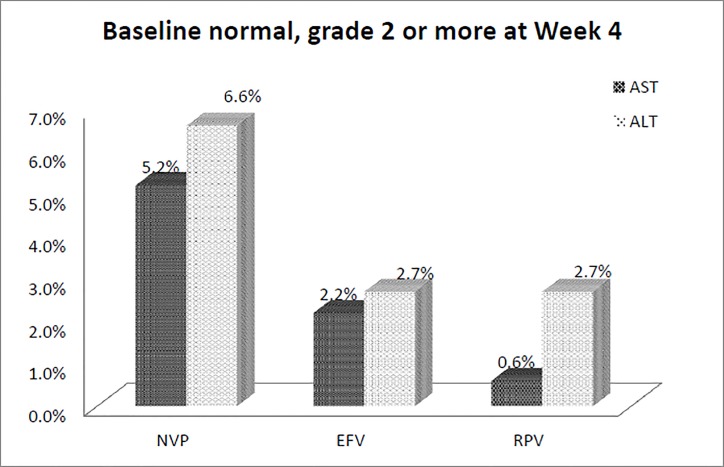
Percentages of grade 2 or higher hepatotoxicity at week 4 in patients with normal aminotransferase levels at baseline (NVP, nevirapine; EFV, efavirenz; RPV, rilpivirine).

Baseline aminotransferase levels available for patients initiating EFV-, NVP-, and RPV-containing regimens are shown in Table 1. Among the 1,455 patients (62.2%) with both baseline and follow-up data of aminotransferases at week 4, 72 (4.9%) patients developed hepatotoxicity of grade 2 or greater: 37 (4.4%) in EFV group, 24 (6.9%) in NVP group and 11 (4.1%) in RPV group. In patients with treatment-emergent hepatic laboratory abnormalities, there was a higher incidence of grade 2 or more AST and ALT elevation in the patients with normal baseline levels of aminotransferase in the NVP group than in the EFV and RPV groups at week 4 (Fig 1).

Of the 24 patients who received NVP with hepatotoxicity, the proportions of HBV coinfection did not differ between those who discontinued and those who continued NVP (7.7% vs. 36.4%, p = 0.084), and neither did the proportions of HCV coinfection (23.1% vs. 45.5%, P = 0.247). Of the 37 patients who received EFV with hepatotoxicity, the proportions of HBV coinfection did not differ between those who discontinued and those who continued EFV (27.3% vs. 34.8%, p = 0.662), and neither did the proportions of HCV coinfection (36.4% vs. 36.0%, P = 0.983) (data not shown).

Univariate analyses of factors associated with hepatotoxicity for all patients are shown in Table 4. We found that older age (*P* = 0.0038), anti-HCV positivity (*P*<0.0001), HBsAg positivity (*P* = 0.0007), and development of skin rashes within 4 weeks of cART (*P =* 0.0008) were associated with hepatotoxicity of grade 2 or greater. In multiple logistic regression analysis, anti-HCV positivity (AOR, 2.865; 95% CI, 1.439–5.704), the development of skin rash (AOR, 2.811; 95% CI, 1.051–7.521) and HBsAg positivity (AOR, 2.397; 95% CI, 1.150–4.997) were independently associated with the development of hepatotoxicity (Tables 5 and 6). Other variables analyzed such as male gender, HIV transmission category, baseline CD4 count and baseline PVL were not statistically significantly associated with hepatotoxicity.

**Table 4:** Univariate analyses for factors associated with hepatotoxicity after initiation of nNRTI-containing regimens within the first 4 weeks.

Variable	With hepatotoxicity	Without hepatotoxicity	ALL	P
	n = 72	n = 1383	n = 1455	
Age, mean (SD), years	36.66 (10.17)	33.46 (9.05)	33.62 (9.13)	0.0038
Male gender, n (%)	70 (97.22)	1320 (95.44)	1390 (95.53)	0.4766
Baseline CD4, mean (SD), cells/μl	262 (157.8)	284 (189.4)	283 (188.0)	0.2616
Baseline CD4 >200 cells/μl, n (%)	48 (67.61)	906 (65.84)	954 (65.93)	0.7599
Baseline plasma HIV RNA load, mean (SD), log_10_ copies/ml	4.71 (0.75)	4.72 (0.75)	4.72 (0.75)	0.889
HIV mono-infected, n (%) [n = 1432]	31 (44.93)	945 (69.33)	976 (68.16)	< .0001
HIV/HBV co-infected, n (%) [n = 1165]	16 (34.04)	173 (15.47)	189 (16.22)	0.0007
HIV/HCV co-infected, n (%) [n = 1298]	27 (46.55)	295 (23.79)	322 (24.81)	< .0001
Development of skin rashes, n (%)	8 (11.11)	47 (3.40)	55 (3.78)	0.0008
Baseline AST, mean (SD), IU/L	39 (23.5)	43 (96.3)	42 (94.0)	0.3726
Baseline ALT, mean (SD), IU/L	43 (29.9)	44 (112.5)	44 (109.7)	0.7984
NVP, n (%)	24 (33.33)	323 (23.36)	347 (23.85)	0.0527
EFV, n (%)	37 (51.39)	801 (57.92)	838 (57.59)	0.2744
RPV, n (%)	11 (15.28)	259 (18.73)	270 (18.56)	0.4629

**Abbreviations:** 95% CI, 95% confidence interval; ALT, alanine aminotransferase; AST, aspartate aminotransferase; EFV, efavirenz; HBV, hepatitis B virus; HCV, hepatitis C virus; nNRTI, non-nucleoside reverse-transcriptase inhibitor; NVP, nevirapine; RPV, rilpivirine; SD, standard deviation.

**Table 5:** Multivariate analyses for factors associated with hepatotoxicity after initiation of nNRTI-containing regimens within the first 4 weeks (HBV/HIV co-infected vs HIV mono-infected).

Variable	Odds Ratio*	95% CI*
Age, per 1-year older	1.025	0.993–1.059
Male gender	-	-
Baseline CD4 count, per 100-cell/μl increase	0.936	0.758–1.155
Baseline PVL, per 1-log_10_ copies/ml increase	1.133	0.695–1.847
HBsAg-positive (vs HIV mono-infected)	2.397	1.150–4.997
Development of skin rashes	2.919	0.976–8.732
Baseline AST, per 1-IU/L increase	0.997	0.985–1.010
Baseline ALT, per 1-IU/L increase	1.000	0.990–1.010
NVP	1.423	0.454–4.453
EFV	0.733	0.251–2.139

* These analyses were conducted in 1455 patients.

**Abbreviations:** 95% CI, 95% confidence interval; ALT, alanine aminotransferase; AST, aspartate aminotransferase

EFV, efavirenz; HBsAg, hepatitis B surface antigen; NVP, nevirapine; PVL, plasma HIV RNA load.

**Table 6:** Multivariate analyses for factors associated with hepatotoxicity after initiation of nNRTI-containing regimens within the first 4 weeks (HCV/HIV co-infected vs. HIV mono-infected).

Variable	Odds Ratio*	95% CI*
Age, per 1-year older	1.008	0.975–1.042
Male gender	2.209	0.502–9.713
Baseline CD4 count, per 100-cell/μl increase	1.041	0.865–1.253
Baseline PVL, per 1-log_10_ copies/ml increase	1.135	0.754–1.707
Anti-HCV-positive (vs HIV mono-infected)	2.865	1.439–5.704
Development of skin rashes	2.811	1.051–7.521
Baseline AST, per 1-IU/L increase	0.998	0.987–1.009
Baseline ALT, per 1-IU/L increase	1.000	0.990–1.010
NVP	1.717	0.710–4.152
EFV	0.861	0.387–1.917

* These analyses were conducted in 1455 patients.

**Abbreviations:** 95% CI, 95% confidence interval; AST, aspartate aminotransferase; ALT, alanine aminotransferase; EFV, efavirenz

HCV, hepatitis C virus; NVP, nevirapine PVL, plasma HIV RNA load.

The results of multivariate analysis for each nNRTI-containing regimen are shown in S2 Table. In univariate analysis in NVP group (data not shown), we found that baseline CD4 cell count (*P* = 0.002), HBsAg positivity (*P* = 0.04) and development of skin rash within 4 weeks of cART (*P*<0.001) were associated with hepatotoxicity of grade 2 or greater. In multiple logistic regression analysis, a higher baseline CD4 cell count (per 100-cell/μl increase, AOR, 1.705; 95% CI, 1.187–2.449) and development of skin rashes (AOR, 4.704; 95% CI, 1.537–14.394) were independently associated with the development of hepatotoxicity.

In univariate analysis in EFV group (data not shown), we found that that older age (*P* = 0.02), anti-HCV-positivity (*P* = 0.02), and HBsAg positivity (*P* = 0.02) were associated with hepatotoxicity of grade 2 or greater. In multiple logistic regression analysis (S2 Table), anti-HCV positivity (AOR, 5.342; 95% CI, 1.865–15.302) and HBsAg positivity (AOR, 3.598; 95% CI, 1.353–9.570) were independently associated with the development of hepatotoxicity. For the patients in RPV group, we were not able to identify any factor statistically significantly associated with hepatotoxicity in either univariate analysis or multiple logistic regression analysis.

## Discussion

In this study conducted in a country where cART comprising 1 nNRTI plus 2 NRTIs remains the preferred regimen for antiretroviral-naïve HIV-positive patients, we found that the overall incidence of hepatotoxicity and skin rashes within 4 weeks of initiation was 4.9% and 14.1%, respectively. HCV coinfection and development of skin rash were independently associated with hepatotoxicity of grade 2 or greater. On the other hand, a higher baseline CD4 cell count and use of NVP plus 2 NRTIs were independently associated with the development of skin rashes.

The rate of skin rashes among HIV-positive patients receiving regimens containing first-generation nNRTIs ranges from 3.8 to 21.6% [7, 17, 28, 33, 38, 39]. In our study, the overall incidence of skin rashes in patients initiating nNRTI-containing regimens was 14.1% (331/2341), which was significantly higher in patients starting NVP-containing regimens (23.7%) than that in those starting EFV-containing regimens (13.2%) and RPV-containing regimens (0.6%). In addition, a higher baseline CD4 count and use of NVP were associated with the development of skin rashes. According to the systematic review and meta-analysis by Shubber et al [10], severe skin rash was more likely to develop among patients on NVP than those on EFV (OR 3.9; 95% CI, 2.5–5.4). In contrast, skin rash was rare in patients receiving RPV in our study, which was similar to the findings observed in the ECHO and THRIVE studies [28]. The mechanism of NVP-associated hypersensitivity is unclear, but several HLA alleles have been found to be associated with NVP hypersensitivity [40, 41]. In our study, the mean onset time of rashes was 18 days among nNRTI users and 22 days among NVP users, which are also consistent with what have been described in the previous reports (8–13 days among nNRTI users [15, 38] and 14–30 days among NVP users) [33, 39].

Hepatotoxicity is observed in 1.1–25.5% of HIV-positive patients treated with cART [7, 14–16, 20–22, 33, 39, 42]. The wide range of hepatotoxicity rates among patients receiving cART may be related to different study designs and populations (age, gender, races, and body weight), prevalence of HBV or HCV coinfection, definitions of hepatotoxicity used, follow-up duration, CD4 counts (particularly in pregnant patients with CD4 >250 cell/μl receiving nevirapine for the prevention of mother-to-children transmission) [43], and antiretroviral regimens initiated. For example, the overall rate of hepatotoxicity, defined as grade 4, was 7.9% in a retrospective cohort study of 560 HIV-positive patients in the Netherlands [42] while the rate of severe hepatotoxicity, defined as grade 3, was 5% in another prospective cohort study of 820 HIV-positive women in 3 countries [39].

The findings that patients on NVP are more likely to develop hepatotoxicity than those on EFV in our study are consistent with those in several studies [10, 14, 15, 18, 25]. Our study also found the baseline CD4 counts and the development of rashes were associated with hepatotoxicity among the patients starting NVP-containing regimens. Likewise, a review of 17 randomized clinical trials of NVP shows rash and other possibly immune-mediated events (most often fever) occurred concurrently with hepatic events in 2.2% of NVP-treated patients, and approximately 46% of symptomatic hepatic events were associated with rash [23]. The key risk factors of this unique rash-associated hepatotoxicity were treatment with NVP, almost exclusively within the first 6 weeks of NVP, and higher baseline CD4 cell counts [23]. Additionally, a 2-fold or greater increase of aminotransferases from the ULN levels was associated with developing rashes in Taiwanese patients receiving NVP-containing regimens [33]. Thus, baseline assessment of liver function is needed in patients who are scheduled to initiate NVP-containing regimens. It is prudent to carefully monitor when NVP-containing regimens is chosen or avoid use of NVP in those who have elevated aminotransferases at baseline.

Higher baseline levels of AST/ALT have been shown to be associated with cART-associated hepatotoxicity [21, 23, 39]. We also found the rate of hepatotoxicity at week 4 was higher in patients with abnormal baseline levels of AST/ALT than that in those with normal baseline levels of AST/ALT (AST, 5.3 vs. 2.6%; ALT, 7.2 vs.3.7%) (data not shown). Chronic viral hepatitis, particularly HCV coinfection, has been recognized a risk factor for hepatotoxicity [16, 18, 21, 23, 25, 26]. Our findings in HIV-positive Taiwanese are in line with the findings of these studies. The mechanism of increased antiretroviral-associated hepatotoxicity in patients with chronic viral hepatitis is not clearly known, but is more likely to be multifactorial. While initiation of antiretroviral therapy containing lamivudine with or without TDF could suppress replication of HBV, previous studies have suggested that hepatic injury may be caused by enhanced HCV replication and cytotoxic T-cell activity during cART-associated immune reconstitution [18, 44, 45]. However, our findings of similar increases of CD4 count within 4 weeks of cART may not support this hypothesis of immune reconstitution-related hepatotoxicity (data not shown).

Our findings may have clinical implications in the management of HIV infection in patients who start cART containing nNRTIs. Monitoring of AST/ALT levels every 2 weeks during the first month of therapy may identify early, and potentially reversible, drug-induced hepatotoxicity, particularly in patients with chronic HCV infection. The appearance of a rash, nausea, or fever during the first 4 weeks of therapy should prompt closer monitoring and assessment.

There are several limitations in this study and interpretation of our findings should be cautious. First, the patients were not randomly assigned to any of the regimens in this cohort and primary care physicians might take into consideration risk behaviors for HIV transmission, baseline liver function, hepatitis coinfection, and immunological as well as virological status of the patients before prescribing any of the 3 nNRTI-containing regimens on an individual basis, which may introduce significant bias or confounding factors. For example, other than the CD4 count cut-offs that are associated with risks for hepatotoxicity related of NVP, we previously found that elevated aminotransferase values at baseline were associated with NVP-associated skin rashes in HIV-positive patients in Taiwan [33]. Clinicians might tend not to prescribe NVP to patients with chronic viral hepatitis or patients who were injection drug users; instead, RPV plus 2 NRTIs was more likely to be used in such populations given the findings that RPV plus 2 NRTIs was associated with lower incidence of hepatotoxicity than EFV in ECHO/THRIVE trials.

Second, we did not have data on exposure to other hepatotoxins (e.g. alcohol and chronic aflatoxin exposure) or agents that might cause hepatotoxicity or skin rashes (e.g. anti-tuberculous agents, fluconazole, trimethoprim/sulfamethoxazole) [46]. Third, because of concerns about the long-term impact of other chronic viral hepatitis, fatty liver and other medications, we limited the observation duration to 4 weeks with an attempt to assess the short-term tolerability of the nNRTI-containing regimens. Such a short observation duration may have underestimated the overall incidence of hepatic and dermatologic complications related to cART in our patients and precluded us from identifying factors associated with chronic elevations of transaminase, such as ongoing exposure to regimens containing ddI, d4T and TDF and short-term exposure to NVP, EFV, FTC and ATV [47]. Fourth, the data regarding the percentages of HBsAg-positive patients with HBeAg-positive and/or detectable HBV DNA and these of HCV-positive patients with detectable HCV RNA were not available. These data might be essential to understanding the role of active replication of HBV or HCV as an underlying cause of hepatotoxicity. Lastly, the case number of patients receive RPV plus 2 NRTIs was much smaller than that of the other two groups (349 patients receiving RPV; 1363 receiving EFV; 629 receiving NVP)

In conclusion, our study among a large treatment-naive HIV-positive population receving nNRTI- containing regimens in Taiwan reveals that the overall rate of hepatotoxicity within 4 weeks of cART initiation was low (4.9%). HCV or HBV coinfection and development of skin rash were independently associated with the development of hepatotoxicity, whereas higher baseline CD4 counts and use of NVP were independently associated with skin rashes within 4 weeks of cART initiation.

## Supporting information

S1 Table Multivariate analyses for factors associated with skin rash after initiation of nNRTI-containing regimens within the first 4 weeks. (DOCX)

S2 Table Multivariate analyses for factors associated with hepatotoxicity after initiation of nNRTI-containing regimens within the first 4 weeks. (DOCX)

S1 Data The minimal data set of the patients in this study. (XLSX)
