# Supplementary material for: Multicenter study of skin rashes and hepatotoxicity in antiretroviral-naïve HIV-positive patients receiving non-nucleoside reverse-transcriptase inhibitor plus nucleoside reverse-transcriptase inhibitors in Taiwan
Source: PLoS One. 2017 Feb 21;12(2):e0171596. doi: 10.1371/journal.pone.0171596 (PMC5319792; doi:10.1371/journal.pone.0171596)
Supplement: S1 Table — (DOCX) [file pone.0171596.s001.docx]

**S1 Table. Multivariate analyses for factors associated with skin rash after initiation of nNRTI-containing regimens within the first 4 weeks.**

|  | **NVP (n=629)** | | **EFV (n=1363)** | |
| --- | --- | --- | --- | --- |
| Variable | Odds Ratio | 95% CI | Odds Ratio | 95% CI |
| Age, per 1-year older | 1.023 | 0.999 - 1.048 | 0.984 | 0.962 - 1.007 |
| Male gender | 0.872 | 0.327 - 2.330 | 0.508 | 0.207 - 1.249 |
| Baseline CD4, per 100-cell/µl increase | 1.000 | 0.675 - 1.482 | 1.001 | 0.824 - 1.216 |
| Baseline CD4 ≥200 cells/µl | 0.742 | 0.306 - 1.798 | 0.829 | 0.406 - 1.695 |
| Baseline CD4 ≥250 cells/µl | 1.287 | 0.630 - 2.627 | 0.570 | 0.278 - 1.168 |
| Baseline CD4 ≥350 cells/µl | 1.419 | 0.582 - 3.461 | 2.326 | 1.211 - 4.466 |
| Baseline plasma HIV RNA load, per 1-log_10_ copies/ml increase | 1.020 | 0.755 - 1.379 | 1.134 | 0.870 - 1.477 |
| HBsAg-positive | 1.335 | 0.658 - 2.711 | 0.818 | 0.479 - 1.397 |
| Anti-HCV-positive | 0.920 | 0.496 - 1.707 | 0.775 | 0.451 - 1.331 |
| Baseline AST, per 1-IU/L increase | 0.999 | 0.992 - 1.006 | 1.000 | 0.996 - 1.004 |
| Baseline ALT, per 1-IU/L increase | 1.000 | 0.993 - 1.008 | 1.000 | 0.997 - 1.003 |

**Abbreviations:** 95% CI, 95% confidence interval; ALT, alanine aminotransferase; AST, aspartate aminotransferase; EFV, efavirenz; HBsAg, hepatitis B surface antigen; HCV, hepatitis C virus; nNRTI, non-nucleoside reverse-transcriptase inhibitor; NVP, nevirapine; SD, standard deviation.
