# Supplementary material for: Multicenter study of skin rashes and hepatotoxicity in antiretroviral-naïve HIV-positive patients receiving non-nucleoside reverse-transcriptase inhibitor plus nucleoside reverse-transcriptase inhibitors in Taiwan
Source: PLoS One. 2017 Feb 21;12(2):e0171596. doi: 10.1371/journal.pone.0171596 (PMC5319792; doi:10.1371/journal.pone.0171596)
Supplement: S2 Table — (DOCX) [file pone.0171596.s002.docx]

**S2 Table. Multivariate analyses for factors associated with hepatotoxicity** **after initiation of nNRTI-containing regimens within the first 4 weeks.**

|  | **NVP (n=347)** | | **EFV (n=822)** | | **RPV (n=270)** | |
| --- | --- | --- | --- | --- | --- | --- |
| Variable | Odds Ratio | 95% CI | Odds Ratio | 95% CI | Odds Ratio | 95% CI |
| Age, per 1-year increase | 0.999 | 0.948- 1.052 | 1.008 | 0.963- 1.055 | 1.051 | 0.974- 1.135 |
| Male gender | 0.471 | 0.072- 3.088 | - | - | - | - |
| Baseline CD4, per 100-cell/µl increase | 1.705 | 1.187-2.449 | 0.920 | 0.721-1.174 | 0.729 | 0.445- 1.192 |
| Baseline PVL, 1-log_10_ copies/ml increase | 1.200 | 0.630- 2.286 | 1.374 | 0.804- 2.349 | 0.644 | 0.203- 2.042 |
| HBsAg-positive (vs HIV mono-infected) | 1.908 | 0.460- 7.914 | 3.598 | 1.353- 9.570 | - | - |
| Anti-HCV-positive (vs HIV mono-infected) | 2.528 | 0.688- 9.293 | 5.342 | 1.865- 15.302 | 1.096 | 0.266- 4.514 |
| Development of skin rashes | 4.704 | 1.537- 14.394 | - | - | - | - |
| Baseline AST, per 1-IU/L increase | 0.969 | 0.930- 1.010 | 0.999 | 0.988- 1.010 | 0.997 | 0.964- 1.030 |
| Baseline ALT, per 1-IU/L increase | 1.007 | 0.992- 1.022 | 0.998 | 0.986- 1.010 | 1.004 | 0.985- 1.022 |

**Abbreviations:** 95% CI, 95% confidence interval; ALT, alanine aminotransferase; AST, aspartate aminotransferase; HBsAg, hepatitis B surface antigen; HCV, hepatitis C virus; nNRTI, non-nucleoside reverse-transcriptase inhibitor; PVL, plasma HIV RNA load; SD, standard deviation.
